# Work-related stress among financial professionals: The impact of age, work experience and education

**DOI:** 10.1371/journal.pone.0314169

**Published:** 2024-11-22

**Authors:** Talgat Kutebayev, Zhanna Utaliyeva, Marina Sautenkova, Gulnara Aizhanova

**Affiliations:** 1 Department of Psychology, L.N. Gumilyov Eurasian National University, Astana, Kazakhstan; 2 Department of Psychology and Special Pedagogy, Aktobe Regional University Named After K. Zhubanov, Aktobe, Kazakhstan; 3 Humanitarian School, Narxoz University, Almaty, Kazakhstan; Universidad Santiago de Cali, COLOMBIA

## Abstract

**Objective:**

The aim of this study was to investigate the differences in the level and sources of work-related stress among financial professionals regarding age, work experience, and educational level.

**Methods:**

A cross-sectional research design was employed among 702 financial professionals in Kazakhstan who worked in different corporate and government organizations. Data on work-related stress were collected via online questionnaires using the Job Stress Survey (JSS).

**Results:**

The results showed that young financial professionals experienced higher stress than older professionals due to the lack of opportunity for advancement. Moreover, the study findings revealed that less experienced financial professionals reported higher levels of stress than more experienced professionals due to the meeting deadlines and conflicts with other departments. The study also found that financial professionals with a postgraduate degree stressed more than professionals with an undergraduate degree on major JSS scales. Furthermore, financial professionals with a postgraduate degree experienced higher stress due to the assignment of disagreeable duties, working overtime, lack of opportunity for advancement, inadequate support by supervisor, dealing with crisis situations, lack of recognition for good work, difficulty getting along with supervisor, insufficient personnel to adequately handle an assignment, lack of participation in policy-making decisions, inadequate salary, excessive paperwork, and covering work for another employee than professionals with undergraduate and vocational degrees.

**Conclusion:**

The findings of this study provide valuable insights for organizations to eliminate and alleviate work-related stress.

## Introduction

According to the World Health Organization, work-related stress is “the response people may have when presented with work demands and pressures that are not matched to their knowledge and abilities and which challenge their ability to cope” [1]. The prevalence of work-related stress in the financial industry has caused serious concerns about the psychological and physical well-being of financial professionals. The American Institute of Stress estimates that stress at work costs the United States industry more than $300 billion a year [2]. It is established that work-related stress results in absenteeism [3–6], turnover [7–9], reduced productivity and employee health problems [10–12]. Approximately one million employees experience daily absences as a result of stress [13,14]. Employees who think about their stressors lose weekly more than 5 hours of office time [15]. The annual healthcare costs due to work-related stress reach $190 billion in the United States [16] and £12 billion in the United Kingdom [17].

Previous research has suggested that age can significantly relate [18], influence [19] and predict levels of work-related stress [20]. The study of Silva & Barreto with 2,054 employees of a large Brazilian government bank revealed that age groups of 40–49 and 50–59 years were both significantly associated with poor self-rated health due to stressful working conditions than 20–29 and 30–39 age groups [21]. Another study with 250 bank workers in Nigeria showed a significant difference between the old and young workers, indicating that the old bank workers were more affected by work-related stress on their productivity than the young workers [22]. Furthermore, the research involving over 2,000 employees aged 50 years or older of an Italian banking group found that organizational aspects of work were associated with traditional stressors such as job demand and lack of job control, as well as relational stressors, including the lack of supervisor support and social support [23]. Moreover, Kan and Yu revealed a significant difference of occupational stress effects on depressive symptoms among age groups. This study with 1239 Chinese bank employees showed that employees over the age of 40 exhibited significantly higher level of depressive symptoms compared to employees aged 30 or below [24].

The relationships of work experience and educational qualification with work-related stress have been the subject of considerable interest. One such study with 320 Zhengzhou Industrial Banking employees revealed that when employees gained experience, their ability to cope with work-related stress also improved [25]. The study with 13,007 people in Germany found that highly educated employees reported higher levels of stress related to the threat of job loss or limited career advancement than less educated employees [26]. Another the study with 370 bankers in Nigeria revealed that work experience of 5 years or less was a significant predictor of high level of stress due to job control, and being in the young age group (35 years or less) was significantly associated with high stress due to control [27]. Similarly, Aderibigbe, Nwokolo & Solomon investigated the impact of work experience and educational qualification on the perceived occupational stress of 1,532 Nigerian graduate employees working in different sectors of the national economy, such as finance, insurance, and commerce [28]. The findings revealed that graduate employees with more work experience showed significantly higher level of occupational stress compared to those with less work experience. Also, the findings showed that educational qualification had a significant effect on occupational stress. Postgraduate employees experienced higher levels of occupational stress than employees with a Bachelor’s Degree and Higher National Diploma. Therefore, work experience and educational qualification can be determining factors of work-related stress experienced by employees [29–31], including financial professionals [18,19,32].

The conceptual framework of this study is Spielberger’s State–Trait Process (STP) model of occupational stress. This model was based on Person-Environment Fit (PE-Fit) and Lazarus’s Transactional Process models and focuses on the perceived severity and frequency of occurrence of two major sources of stress: job pressures and lack of support [33]. In comparison to other models of occupational stress, the STP model places a more significant focus on how individual differences in personality traits influence the perception and appraisal of workplace stressors [34]. The STP model of occupational stress is comprised of three fundamental elements. These elements include sources of job stress, employee’s perception and appraisal of a specific stressor, and emotional responses elicited by the perception and appraisal of a stressor as threatening [33]. Based on Spielberger’s STP model, the extensively used Job Stress Survey (JSS) [35,36] assesses the perceived severity and frequency of specific sources of occupational stress experienced by employees in the workplace, relating to job pressures (e.g., excessive paperwork, meeting deadlines) and lack of support (e.g., lack of opportunity for advancement, poor or inadequate supervision) [37].

Considering the above mentioned studies on the relationships between age, work experience, educational qualification, and work-related stress, limited attention was paid to the differences in job stress regarding these demographic variables. The financial industry is characterized by its intense competition [38,39], challenging conditions and demanding work environment [40,41], where professionals are often exposed to work-related stress. Understanding the relationships between age, work experience, educational background and the level and sources of stress experienced by financial professionals is essential to develop effective interventions and strategies, enabling organizations and policymakers to provide support, improve working conditions, and enhance overall health and productivity. To fill the current gap, this study aims to investigate the differences in the level and sources of work-related stress among financial professionals regarding age, work experience, and educational level. The Job Stress Survey [35,36] was used to achieve the objectives of this study and to measure work-related stress among Kazakhstani financial employees.

## Method

### Research design

A cross-sectional design was employed among financial professionals in Kazakhstan. The study took over 3 months, from April 4 to July 12, 2022. Human Resources departments of the financial organizations were emailed and invited to participate in this study. The online survey to assess work-related stress was distributed within the organizations by HR departments. After providing written informed consent, participants completed the online survey. Before starting to answer the questionnaire, employees were instructed about the general rules and how to respond to the JSS items, as well as the significance of the research and its contribution to the financial workplace. Participants were informed that all their responses would be anonymous and confidential, participation was completely voluntary and participants could leave the study at any time. Participants spent approximately 15 to 20 minutes completing the online survey and without any financial incentives. The study was approved by the Social Sciences Research Ethics Committee of the L.N. Gumilyov Eurasian National University, Astana, Kazakhstan, in accordance with the 1964 Helsinki Declaration (approval number EP_22-23/7).

### Participants

Totally, 702 employees were invited to participate in this study. The study comprised 234 men, aged 21–63 years (mean age = 34.5, *SD* = 8.4) and 468 women, aged 21–65 years (mean age = 40.7, *SD* = 9.5). The age of the participants ranged from 21 to 65 years, with a mean age of 38.7 (*SD* = 9.61) for the whole sample.

### Measures

#### Demographic information

Participants provided sociodemographic information, including age, gender, highest level of education (secondary vocational education, bachelor’s degree, master’s degree, doctoral degree, other), type of employment (full-time or part-time), length of work experience, and marital status (married, single, separated, divorced, widowed, other).

#### Job stress

The Job Stress Survey (JSS) [35,36] was used to assess generic sources of work-related stress experienced by employees and measure components of work stress associated with the job itself, with supervisors, colleagues, or the regulations and processes of the organization. The Job Stress Survey was developed by Spielberger [42] and his colleagues in the middle of the 1980s [43,44].

The JSS instrument consists of 30 items describing 30 potentially stressful work-related situations (e.g., “inadequate salary”, “working overtime”). Participants were asked to respond to these items by choosing the perceived severity and frequency of occurrence of each stressor event. Firstly, the participants rated each stressor event in terms of its perceived severity on a 9-point scale, ranging from 1 (low stress) to 9 (high stress). After completion of the first part, the participants proceeded to the second part, the frequency of experiencing each stressor event during the past year and rated on the same nine-point scale.

Job Stress Index was used to assess the overall level of stress based on the combined average ratings of perceived severity and frequency of occurrence of the 30 stressor events. Components of work-related stress associated with the job itself were assessed by the Job Pressure subscale, consisting of ten items, such as “dealing with crisis situations”, “making critical on-the-spot decisions”, and “excessive paperwork”. The lack of support from supervisors, colleagues, or organizational regulations and processes was measured by the Lack of Organizational Support subscale, comprising ten items, such as “fellow workers not doing their jobs”, “lack of participation in policy-making decisions”, and “inadequate support by supervisor”.

In this research, the Job Stress Index total score demonstrated a high internal consistency reliability, with a Cronbach’s α of = .98. Similarly, the Job Pressure subscale and the Lack of Organizational Support subscale showed high levels of internal consistency reliability, each with a Cronbach’s α of = .95.

#### Data analyses

Descriptive statistics (frequencies, percentages, means, standard deviations, skewness and kurtosis) were calculated for all variables. To assess the validity and internal consistency reliability of the JSS instrument, Cronbach’s alpha coefficients were used. Analyses of variance (ANOVAs) and Welch F-tests were conducted to investigate and determine statistically significant differences in work-related stress between age groups, work experience, and educational level. Eta-squared (η^2^) and *β* coefficient were calculated to identify effect size. Pearson Correlations were used to examine the associations between study variables, with statistical significance at a p-value < .05. Multiple linear regression analyses were performed to determine predictions and percentages of variance explained. All data were analyzed using IBM SPSS version 27.

## Results

The normality of the dataset was measured by the skewness and kurtosis tests. The skewness of job stress was found to be 1.54, job pressure = 1.37, lack of organizational support = 1.68, age = .25, educational level = .91, indicating that the distributions were right-skewed; and work experience was found to be -.279, showing that the distribution was left-skewed. Positive kurtosis values were found for job stress = 2.71, job pressure = 1.74, lack of organizational support = 3.27, and educational level = 1.54, indicating leptokurtic distributions, while negative kurtosis values for age = -.92 and work experience = -.99 suggested a platykurtic distribution. Multicollinearity was assessed by the Variance Inflation Factor (VIF) values. The results revealed the following VIF values for age = 5.28, work experience = 4.33, and educational level = 1.99. In this study, the data had normal distributions, ranging from −2 to +2 for skewness and ‐7 to +7 for kurtosis [45,46]. No multicollinearity was detected due to low values of VIF (<10) [47]. Therefore, the dataset was used in further analyses.

### Participant characteristics

Regarding age, no participant was younger than 21 years, 162 of the participants were between 21 and 30 years (23%), 259 were between 31 and 40 (37%), 186 were between 41 and 50 (26%), and 95 were over 51 (14%). 422 of the participants were married (60%), 201 were unmarried (29%), and 79 participants indicated being separated, divorced or widowed (11%). Regarding educational level, 567 participants had a bachelor’s degree (81%), 117 had a postgraduate degree (17%), and 18 had a vocational degree (2%). As for employment, 99.3% of the participants had full-time work and the mean duration was 15 years. Concerning work experience, most participants had 11–20 years of experience (n = 251), 5–10 years (n = 171), < 5 years (n = 105), and > 20 years (n = 175).

### Correlational and multiple linear regression analyses

The results of the correlation analyses are provided in Table 1. Age and work experience were negatively associated with job stress, job pressure and lack of support, and the correlations were not statistically significant. However, educational level was significantly and positively associated with job stress, job pressure and lack of support.

**Table 1:** Pearson correlations of age, work experience, educational level with job stress, job pressure, lack of support.

Variable	Job stress	Job pressure	Lack of support
**Age**	-.04	-.02	-.02
**Work experience**	-.04	-.04	-.01
**Educational level**	.11**	.09*	.11**

Note.

*p < .05

**p < .01

***p < .001.

The results of multiple linear regressions of age, work experience, and educational level with job stress, job pressure and lack of organizational support are provided in Table 2. The overall regression model for job stress was statistically significant (R^2^ = .03, F (3, 698) = 6.92, p = < .001). It was found that work experience significantly and negatively predicted job stress, while educational level was a significant positive predictor of job stress, however, age did not significantly predict job stress. The regression model for job pressure was statistically significant (R^2^ = .03, F (3, 698) = 7.26, p = < .001). It was revealed that educational level significantly and positively predicted job pressure. Regarding age and work experience, no significant predictions of job pressure were observed. The results of regression indicated that the model for lack of organizational support was statistically significant (R^2^ = .02, F (3, 698) = 5.46, p = .001). Work experience showed to be a significant negative predictor of lack of support, and educational level had a significant positive prediction. However, age demonstrated no significant prediction of lack of organizational support.

**Table 2:** Multiple linear regressions of age, work experience, educational level with JSS scales.

	*R*	*R* ^*2*^	*Adj*. *R*^*2*^	*F*	*P*	*β*	*P*
***Job stress***	.17	.03	.03	6.92	**< .001**		< .001
Age						-.05	.538
Work experience						-.18	**.023**
Educational level						.13	**.011**
***Job pressure***	.17	.03	.03	7.26	**< .001**		< .001
Age						-.09	.297
Work experience						-.15	.057
Educational level						.12	**.022**
***Lack of support***	.15	.02	.02	5.46	**.001**		< .001
Age						-.04	.644
Work experience						-.16	**.036**
Educational level						.13	**.015**

*Note*. *β* = *β* coefficient effect size; small: *β*≥0.10; medium: *β*≥0.30; large: *β*≥0.50 [48].

### Age, work experience and educational level differences in work-related stress

Descriptive statistics and results of analyses of variance (ANOVAs) on age differences for the Job Stress Survey (JSS) scales are provided in Table 3. Among age groups (21–30 years, 31–40 years, 41–50 years and 51+ years), young financial professionals (below 30) experienced the highest stress on the JSS Severity and Index subscales, except Frequency subscale, which is the highest among 31–40 age group. Regarding the Job Pressure Frequency and Job Pressure Index subscales, professionals of the middle age group (31–40) reported the highest levels of stress, besides the Job Pressure Severity subscale, which is the highest among 21–30 age group. As for the Lack of Organizational Support subscale, young financial professionals (below 30) experienced the highest stress on the Severity subscale, professionals of the middle age group (31–40) experienced stress more often on the Frequency subscale, and professionals aged 41–50 years experienced the highest stress on the Index subscale, while older professionals (above 51) experienced the least level of stress; nevertheless, all differences were not statistically significant. However, on the item level, statistically significant differences among financial professionals were observed for the lack of opportunity for advancement (see also S1 Table in S4 File). Games-Howell post hoc tests revealed significant differences between age groups of 21–30 years, 31–40 years, and 51+ years so that professionals aged 21 to 30 years experienced higher stress due to the lack of opportunity for advancement (small effect size).

**Table 3:** Descriptive statistics and results of ANOVAs on age differences for JSS scales.

Age group (years)	21–30 (n = 162)		31–40 (n = 259)		41–50 (n = 186)		51+ (n = 95)		*F/ Welch’s F*	*p*	*η* ^*2*^
	*M*	*SD*	*M*	*SD*	*M*	*SD*	*M*	*SD*			
***Total Stress***											
Severity	3.67	1.84	3.45	1.55	3.39	1.68	3.31	1.60	1.22	.303	.005
Frequency	3.11	1.59	3.19	1.47	3.14	1.66	2.92	1.52	.676	.567	.003
Index	15.93	13.45	15.66	12.86	15.53	14.42	14.13	13.02	.393	.758	.002
***Job Pressure***											
Severity	4.05	1.94	3.84	1.71	3.80	1.75	3.82	1.86	.693	.557	.003
Frequency	3.47	1.77	3.64	1.71	3.52	1.78	3.44	1.86	.501	.682	.002
Index	18.56	15.35	18.58	15.13	17.91	15.67	17.74	15.51	.124	.946	.001
***Lack of Support***											
Severity	3.49	1.93	3.37	1.72	3.31	1.88	3.24	1.72	.482	.695	.002
Frequency	2.92	1.63	3.09	1.62	3.05	1.83	2.83	1.62	.753	.521	.003
Index	14.43	13.24	14.59	13.60	14.77	15.36	12.86	12.56	.448	.719	.002
***Item 3 Index*. *Lack of opportunity for advancement***	20.29_a_	24.08	19.19_a_	23.00	15.91_b_	19.69	13.13_a_	17.68	3.47	**.017**	.013

*Note*. Welch’s F test was performed with the item 3. Value in bold is statistically significant at the p<0.05 level. Means with the same subscript are significantly different at *p* < .05 in Games-Howell post hoc tests. η^2^ = eta-squared effect size; small: η^2^≥0.01; medium: η^2^≥0.06; large: η^2^≥0.14 [48].

Descriptive statistics and results of ANOVAs on work experience differences for JSS scales are provided in Table 4. Financial professionals with less than 5 years of work experience scored the highest stress on all JSS subscales than professionals with 5–10, 11–20 and more than 20 years of experience; nonetheless, there were no statistically significant differences. But item analysis revealed statistically significant differences among financial professionals such as meeting deadlines and conflicts with other departments (see also S2 Table in S4 File). Games-Howell post hoc tests found significant differences between professionals with less than 5 years of work experience and professionals with 11–20 years of experience so that professionals with less than 5 years of work experience reported higher level of stress due to the meeting deadlines (small effect size). Also, financial employees with 5–10 years of work experience rated conflicts with other departments as more stressful than employees with 11–20 years of experience (small effect size).

**Table 4:** Descriptive statistics and results of ANOVAs on work experience differences for JSS scales.

Work Experience	< 5 years (n = 105)		5–10 years (n = 171)		11–20 years (n = 251)		> 20 years (n = 175)		*F/ Welch’s F*	*p*	*η* ^*2*^
	*M*	*SD*	*M*	*SD*	*M*	*SD*	*M*	*SD*			
***Total Stress***											
Severity	3.74	1.91	3.55	1.70	3.29	1.50	3.47	1.67	2.10	.099	.009
Frequency	3.23	1.73	3.17	1.48	3.05	1.47	3.12	1.65	.408	.747	.002
Index	16.99	14.80	15.82	13.18	14.42	12.07	15.75	14.60	1.02	.381	.004
***Job Pressure***											
Severity	4.18	1.97	3.97	1.84	3.65	1.65	3.91	1.84	2.54	.056	.011
Frequency	3.67	1.89	3.60	1.70	3.43	1.66	3.57	1.88	.595	.619	.003
Index	20.22	16.41	18.85	15.56	16.78	13.86	18.71	16.41	1.48	.218	.006
***Lack of Support***											
Severity	3.57	2.04	3.42	1.82	3.20	1.67	3.42	1.85	1.22	.303	.005
Frequency	3.10	1.77	2.99	1.61	2.94	1.59	3.06	1.82	.283	.837	.001
Index	15.45	14.88	14.38	13.37	13.48	12.82	14.98	15.12	.666	.573	.003
***Item 26 Index*. *Meeting deadlines***	31.06_a_	28.06	26.93_b_	25.74	22.38_a_	23.09	24.45_c_	23.87	3.05	**.029**	.014
***Item 30 Index*. *Conflicts with other department***	9.74_b_	14.07	10.93_a_	15.89	7.07_a_	11.18	10.27_c_	17.64	3.54	**.015**	.012

*Note*. Welch’s F test was performed with the items 26 and 30. Values in bold are statistically significant at the p<0.05 level. Means with the same subscript are significantly different at *p* < .05 in Games-Howell post hoc tests. η^2^ = eta-squared effect size; small: η^2^≥0.01; medium: η^2^≥0.06; large: η^2^≥0.14 [48].

Descriptive statistics and results of ANOVAs on educational level differences for JSS scales are provided in Table 5. Games-Howell post hoc tests revealed significant differences between professionals with an undergraduate degree and professionals with a postgraduate degree so that financial professionals with a postgraduate degree experienced higher levels of stress on all JSS subscales than professionals with an undergraduate degree. Financial professionals with a postgraduate degree experienced more severe stress according to the JSS Severity, Job Pressure Severity and Lack of Organizational Support Severity subscales (small-to-medium effect size). Also, they experienced more frequent episodes of stress according to the JSS Frequency, Job Pressure Frequency and Lack of Organizational Support Frequency subscales (small effect size), as well as more overall stress according to the JSS Index, Job Pressure Index and Lack of Organizational Support Index subscales (small effect size) than professionals with an undergraduate degree [49].

**Table 5:** Descriptive statistics and results of ANOVAs on educational level differences for JSS scales.

Educational Level	Vocational degree		Undergraduate degree		Postgraduate degree		*F/ Welch’s F*	*p*	*η* ^*2*^
	(n = 18)		(n = 567)		(n = 117)				
	*M*	*SD*	*M*	*SD*	*M*	*SD*			
***Total Stress***									
Severity	3.50_b_	1.69	3.36_a_	1.64	4.16_a_	1.68	10.92	**.001**	.032
Frequency	3.33_b_	1.72	3.04_a_	1.58	3.56_a_	1.51	5.76	**.006**	.016
Index	17.06_b_	16.24	14.65_a_	13.20	19.28_a_	13.46	5.79	**.006**	.017
**Job Pressure**									
Severity	4.11_b_	1.88	3.74_a_	1.78	4.50_a_	1.76	9.23	**.001**	.026
Frequency	3.78_b_	1.99	3.44_a_	1.75	3.99_a_	1.69	5.07	**.011**	.014
Index	20.61_b_	17.43	17.41_a_	15.13	22.13_a_	15.58	4.59	**.016**	.014
**Lack of Support**									
Severity	3.50_b_	1.76	3.22_a_	1.77	4.04_a_	1.87	9.48	**.001**	.029
Frequency	3.33_b_	1.82	2.91_a_	1.66	3.44_a_	1.67	5.11	**.010**	.015
Index	15.94_b_	15.46	13.46_a_	13.45	18.55_a_	14.85	5.91	**.005**	.019
***Item 1 Index*. *Assignment of disagreeable duties***	18.00_b_	18.10	15.06_a_	17.57	20.23_a_	18.47	3.93	**.027**	.012
***Item 2 Index*. *Working overtime***	17.44_b_	18.52	15.23_a_	20.17	24.73_a_	24.14	7.81	**.001**	.028
***Item 3 Index*. *Lack of opportunity for advancement***	15.17_a_	13.66	16.33_a_	21.09	25.05_a_	25.05	6.33	**.004**	.022
***Item 6 Index*. *Inadequate support by supervisor***	13.06_b_	19.12	14.17_a_	18.86	22.95_a_	23.73	7.09	**.002**	.027
***Item 7 Index*. *Dealing with crisis situations***	19.11_b_	12.27	16.13_a_	18.30	21.00_a_	19.73	3.26	**.048**	.010
***Item 8 Index*. *Lack of recognition for good work***	27.28_b_	28.89	15.42_a_	19.90	21.25_a_	20.93	5.01	**.011**	.018
***Item 13 Index*. *Difficulty getting along with supervisor***	7.28_a_	8.11	11.04_b_	17.13	15.53_a_	19.92	4.83	**.012**	.011
***Item 15 Index*. *Insufficient personnel to adequately handle an assignment***	12.00_b_	18.38	11.79_a_	16.70	16.98_a_	18.84	3.77	**.031**	.013
***Item 18 Index*. *Lack of participation in policy-making decisions***	13.56_b_	19.19	11.14_a_	15.81	16.11_a_	18.52	3.68	**.034**	.013
***Item 19 Index*. *Inadequate salary***	17.56_b_	24.81	17.95_a_	24.06	24.97_a_	25.87	3.62	**.035**	.011
***Item 25 Index*. *Excessive paperwork***	18.11_b_	29.62	17.37_a_	20.88	25.53_a_	24.48	5.57	**.007**	.019
***Item 28 Index*. *Covering work for another employee***	14.67_b_	20.48	15.48_a_	19.87	22.19_a_	21.71	4.76	**.014**	.015

*Note*. Welch’s F test was performed with Total Stress, Job Pressure, Lack of Support Scales, and items 1, 2, 3, 6, 7, 8, 13, 15, 18, 19, 25 and 28. Values in bold is statistically significant at the p<0.05 level. Means with the same subscript are significantly different at *p* < .05 in Games-Howell post hoc tests. η^2^ = eta-squared effect size; small: η^2^≥0.01; medium: η^2^≥0.06; large: η^2^≥0.14 [48].

On the item level, financial professionals holding a postgraduate degree reported significantly higher stress due to the assignment of disagreeable duties, dealing with crisis situations, lack of recognition for good work, insufficient personnel to adequately handle an assignment, lack of participation in policy-making decisions, inadequate salary, excessive paperwork, covering work for another employee (small effect size), as well as working overtime and inadequate support by supervisor (small-to-medium effect size) than professionals with an undergraduate degree [49]. Also, professionals with a postgraduate degree reported higher stress due to the lack of opportunity for advancement than professionals with undergraduate and vocational degrees (small effect size). Moreover, professionals with a postgraduate degree reported higher stress due to the difficulty getting along with supervisor than professionals with a vocational degree (small effect size) (see also S3 Table in S4 File).

## Discussion

The present study aimed to investigate the differences in the level and sources of work-related stress among financial professionals regarding age, work experience, and educational level. The findings of this study clearly demonstrate that work-related stress in this particular occupational group is influenced by these factors. Regarding age differences on major JSS scales, the results of this study revealed no significant predictions and differences [50–52], except item level, so that young financial professionals (below 30) experienced higher stress due to the lack of opportunity for advancement than professionals of 31–40 and 51+ age groups [27]. The differences found between age groups may be explained by increased competition in financial settings, less experience and human resources policies relating to the limited opportunities for advancement that young financial professionals encounter [53,54]. Moreover, they are more sensitive and more likely to face work-related stress [55].

As to work experience differences on all JSS subscales, the study did not find statistically significant differences [56,57]. However, work experience significantly and negatively predicted job stress and lack of support so that financial professionals with less than 5 years of work experience scored the highest stress than professionals with 5–10, 11–20 and more than 20 years of experience. Moreover, item analysis revealed that financial professionals with less than 5 years of work experience reported higher level of stress due to the meeting deadlines than professionals with 11–20 years of experience [27]. This may be related to the low experience of new employees compared to more experienced and competent professionals [54]. Furthermore, financial employees with 5–10 years of work experience rated conflicts with other departments as more stressful than employees with 11–20 years of experience, suggesting a potential relationship with intergroup conflict. Intergroup conflicts occur as a result of differing interests, goals, and principles. Such factors as competition, the intention to control key services, and misconceptions may also contribute to this conflict; thus, negatively influencing the organization’s overall performance. The resolution and timely mitigation of intergroup conflicts are essential to prevent organizations from negative outcomes and serious consequences [58,59].

The current study found significant educational level differences among financial professionals on major JSS scales–total stress, job pressure and lack of organizational support, as well as significant negative predictions, so that financial professionals with a postgraduate degree experienced higher levels of stress than professionals with an undergraduate degree [32,36,60,61]. Also, differences were found on the item level, financial professionals with a postgraduate degree experienced higher stress due to the assignment of disagreeable duties, dealing with crisis situations, lack of recognition for good work, insufficient personnel to adequately handle an assignment, lack of participation in policy-making decisions, inadequate salary, excessive paperwork, covering work for another employee, working overtime, and inadequate support by supervisor than professionals with an undergraduate degree [26]. Besides, professionals with a postgraduate degree showed higher stress due to the lack of opportunity for advancement than professionals with undergraduate and vocational degrees [26]. Moreover, professionals with a postgraduate degree reported higher stress due to the difficulty getting along with supervisor than professionals with a vocational degree. Several possible reasons may explain why financial professionals with a postgraduate degree experience higher stress than professionals with undergraduate and vocational degrees. High job demands, more responsibilities, and being involved in critical decision-making can lead to increased levels of stress and additional pressure [62–64]. They may have higher career ambitions, intentions to hold key positions in an organization, and the competition for career advancement can be intense [26,65,66]. They may also face challenges in balancing work and personal life, especially if their roles require working long hours.

The findings of this study provide valuable insights for organizations to eliminate and alleviate work-related stress regarding age, work experience, and educational level, as well as to create a non-judgmental and supportive work environment for the employees to manage work stress effectively. Consequently, financial organizations and employers may consider these factors and make effective interventions to reduce work-related stress. Regarding organizational support, workplace mentoring and career development programs should be implemented to provide young employees clearer growth guidance [67,68]. Organizations can offer time management trainings to help less experienced employees manage deadlines effectively [69]. Additionally, to prevent conflicts with different departments, conflict resolution workshops could be organized to improve communication and relationships among employees, as well as cross-departmental collaboration through regular meetings and joint projects [58,70,71]. Human resource policies can be adapted to address the unique stressors experienced by financial professionals with a postgraduate degree. This can include changes in the career advancement policy to provide a clear guide for growth, and also considerable modifications of the decision-making processes to improve employees’ participation. Organizations can allow employees some control over their task schedule, so they can choose when to complete more challenging ones and ensure that the assigned duties are not disagreeable. Regular leadership training programs for supervisors are needed to enhance their managerial skills and to provide constructive feedback that supports and motivates employees [72–76]. The compensation payment for average job performance may help to address the issues of inadequate salary and lack of recognition for good work. Companies can regularly assess employees’ work productivity and hire additional staff to mitigate job stress associated with insufficient personnel and covering work for another employee. These solutions have also been mentioned to avoid banking crises [77]. In addition, implementing stress management programs, such as Stress Inoculation Training and the work-related stress model-based Workplace Mental Health Promotion Program, as well as promoting work-life balance, and fostering a culture of open communication about stress and mental health can contribute to a healthier and more resilient workplace within the financial profession [78–85].

The study had several limitations. First, the cross-sectional design was employed, which limits the explanation of causal relationships among the variables. Regarding the findings of differences in the level and sources of work-related stress among financial professionals, a longitudinal design might explain the relationships between the study’s variables and how job stressors evolve and change over time. Second, the online survey was distributed only in Russian, while Russian was a second language for some participants. This might have affected how the participants answered the questionnaires due to misconceptions and improper interpretations.

In conclusion, the differences in age and work experience were not found on major JSS scales but were found on the item level so that lack of opportunity for advancement stressed young financial professionals more than older professionals, and less experienced professionals experienced higher stress due to the meeting deadlines and conflicts with other departments than more experienced professionals. The differences in educational level were found on major JSS scales so that financial professionals with a postgraduate degree stressed more than professionals with an undergraduate degree. Also, differences were found on the item level, financial professionals with a postgraduate degree experienced higher stress due to the assignment of disagreeable duties, working overtime, lack of opportunity for advancement, inadequate support by supervisor, dealing with crisis situations, lack of recognition for good work, difficulty getting along with supervisor, insufficient personnel to adequately handle an assignment, lack of participation in policy-making decisions, inadequate salary, excessive paperwork and covering work for another employee than professionals with undergraduate and vocational degrees. This study facilitates a better understanding of the sources of work-related stress among financial professionals, thus emphasizing potential ways for eliminating and alleviating. Preventive interventions may substantially decrease work-related stress and address associated factors, including staff turnover and absenteeism, while also improving productivity, physical health and mental well-being of employees. Future studies, exploring age, work experience and educational level differences among financial professionals in other countries may be required to confirm the results.

## Supporting information

S1 File Dataset of age. (XLSX)

S2 File Dataset of work experience. (XLSX)

S3 File Dataset of education. (XLSX)

S4 File Supplementary tables. (DOCX)
