# Supplementary material for: Work-related stress among financial professionals: The impact of age, work experience and education
Source: PLoS One. 2024 Nov 22;19(11):e0314169. doi: 10.1371/journal.pone.0314169 (PMC11584122; doi:10.1371/journal.pone.0314169)
Supplement: S4 File — (DOCX) [file pone.0314169.s004.docx]

**Supplementary Table 1**

*Descriptive Statistics and Results of ANOVAs on Age Differences for all JSS Items*

| **Age group**  **(years)** | **21-30**  (n = 162) | | **31-40**  (n = 259) | | **41-50**  (n = 186) | | **51+**  (n = 95) | |  |  |  |
| --- | --- | --- | --- | --- | --- | --- | --- | --- | --- | --- | --- |
|  | ***M*** | ***SD*** | ***M*** | ***SD*** | ***M*** | ***SD*** | ***M*** | ***SD*** | ***F/*** ***Welch’s F*** | ***p*** | ***η^2^*** |
| Assignment of disagreeable duties | 17.25 | 19.33 | 15.67 | 16.47 | 16.10 | 19.16 | 14.53 | 16.00 | .514 | .672 | .002 |
| Working overtime | 19.02 | 22.28 | 17.73 | 21.44 | 15.66 | 20.93 | 13.26 | 17.99 | 1.84 | .139 | .008 |
| **Lack of opportunity for advancement** | 20.29 | 24.08 | 19.19 | 23.00 | 15.91 | 19.69 | 13.13 | 17.68 | 3.47 | **.017** | .013 |
| Assignment of new or unfamiliar duties | 15.67 | 16.56 | 18.48 | 20.18 | 17.82 | 21.15 | 16.00 | 21.30 | .848 | .468 | .004 |
| Fellow workers not doing their jobs | 15.48 | 17.90 | 15.22 | 19.76 | 14.48 | 17.82 | 13.64 | 18.44 | .252 | .860 | .001 |
| Inadequate support by supervisor | 15.27 | 19.31 | 15.46 | 20.17 | 16.64 | 20.85 | 14.51 | 19.22 | .280 | .840 | .001 |
| Dealing with crisis situations | 16.10 | 16.94 | 17.44 | 18.97 | 18.06 | 19.96 | 15.40 | 16.73 | .616 | .605 | .003 |
| Lack of recognition for good work | 13.88 | 16.60 | 17.82 | 21.19 | 17.17 | 22.08 | 17.52 | 21.26 | 1.36 | .253 | .006 |
| Performing tasks not in job description | 15.68 | 18.68 | 13.43 | 16.42 | 15.63 | 19.57 | 15.89 | 18.40 | .864 | .459 | .004 |
| Inadequate or poor quality equipment | 18.67 | 24.06 | 15.46 | 19.53 | 14.40 | 19.02 | 13.43 | 18.26 | 1.80 | .146 | .008 |
| Assignment of increased responsibility | 24.15 | 23.49 | 27.85 | 26.44 | 26.01 | 24.58 | 28.49 | 26.50 | .928 | .427 | .004 |
| Periods of inactivity | 10.72 | 13.70 | 9.73 | 14.04 | 9.48 | 13.99 | 8.71 | 12.91 | .469 | .704 | .002 |
| Difficulty getting along with supervisor | 11.91 | 15.83 | 11.56 | 17.59 | 12.82 | 20.21 | 9.48 | 14.27 | .769 | .512 | .003 |
| Experiencing negative attitudes toward the organization | 10.11 | 15.99 | 9.08 | 16.00 | 11.41 | 19.55 | 7.89 | 12.67 | 1.17 | .319 | .005 |
| Insufficient personnel to adequately handle an assignment | 12.90 | 17.48 | 11.68 | 15.90 | 13.77 | 18.20 | 12.74 | 18.22 | .549 | .649 | .002 |
| Making critical on-the-spot decisions | 18.11 | 19.56 | 18.83 | 21.18 | 18.20 | 21.38 | 17.46 | 18.70 | .115 | .951 | .001 |
| Personal insult from customer / consumer / colleague | 13.34 | 19.24 | 11.17 | 16.91 | 10.84 | 18.97 | 9.24 | 17.09 | 1,15 | .330 | .005 |
| Lack of participation in policy-making decisions | 12.23 | 17.06 | 12.20 | 16.59 | 12.24 | 16.18 | 10.80 | 15.84 | .203 | .894 | .001 |
| Inadequate salary | 19.93 | 23.99 | 18.54 | 24.61 | 20.40 | 25.24 | 16.77 | 23.70 | .567 | .637 | .002 |
| Competition for advancement | 14.97 | 21.17 | 14.75 | 20.29 | 13.74 | 20.93 | 10.69 | 16.65 | 1.10 | .348 | .005 |
| Poor or inadequate supervision | 9.20 | 14.56 | 10.35 | 17.00 | 14.16 | 20.49 | 11.67 | 20.27 | 2.56 | .054 | .011 |
| Noisy work area | 12.27 | 18.18 | 14.23 | 21.06 | 13.56 | 20.63 | 10.28 | 20.02 | 1.01 | .386 | .004 |
| Frequent interruptions | 14.64 | 20.39 | 14.31 | 19.24 | 14.24 | 19.29 | 13.44 | 19.48 | .077 | .973 | .001 |
| Frequent changes from boring to demanding duties | 13.72 | 18.14 | 13.28 | 18.08 | 13.00 | 18.04 | 10.27 | 14.72 | .858 | .462 | .004 |
| Excessive paperwork | 21.24 | 23.04 | 18.13 | 21.32 | 17.33 | 21.03 | 18.94 | 23.41 | 1.03 | .380 | .004 |
| Meeting deadlines | 28.47 | 27.56 | 25.45 | 24.18 | 22.90 | 23.23 | 24.20 | 24.68 | 1.53 | .207 | .007 |
| Insufficient personal time | 17.41 | 23.20 | 18.01 | 22.07 | 15.96 | 21.74 | 16.67 | 23.61 | .321 | .811 | .001 |
| Covering work for another employee | 18.46 | 22.17 | 16.69 | 20.11 | 15.67 | 19.37 | 14.84 | 19.51 | .822 | .482 | .004 |
| Poorly motivated coworkers | 16.79 | 20.37 | 19.42 | 22.61 | 18.52 | 21.74 | 15.57 | 20.27 | .981 | .401 | .004 |
| Conflicts with other department | 9.93 | 13.70 | 8.49 | 13.59 | 9.91 | 16.75 | 8.55 | 14.83 | .543 | .653 | .002 |

*Note.* Welch’s F test was performed to identify age differences. η^2^ = eta-squared effect size; small: η^2^≥0.01; medium: η^2^≥0.06; large: η^2^≥0.14 [1].

**Supplementary Table 2**

*Descriptive Statistics and Results of ANOVAs on Work Experience Differences for all JSS Items*

| **Work Experience** | **< 5 years**  (n = 105) | | **5-10 years**  (n = 171) | | **11-20 years**  (n = 251) | | **> 20 years**  (n = 175) | |  |  |  |
| --- | --- | --- | --- | --- | --- | --- | --- | --- | --- | --- | --- |
|  | ***M*** | ***SD*** | ***M*** | ***SD*** | ***M*** | ***SD*** | ***M*** | ***SD*** | ***F/ Welch’s F*** | ***p*** | ***η^2^*** |
| Assignment of disagreeable duties | 18.33 | 20.56 | 15.10 | 16.72 | 15.63 | 17.45 | 15.99 | 17.66 | .781 | .505 | .003 |
| Working overtime | 19.66 | 23.30 | 17.30 | 21.11 | 16.51 | 20.76 | 15.31 | 20.21 | .975 | .404 | .004 |
| Lack of opportunity for advancement | 20.64 | 24.60 | 18.61 | 22.18 | 17.41 | 21.67 | 15.69 | 19.99 | 1.24 | .294 | .005 |
| Assignment of new or unfamiliar duties | 19.96 | 20.74 | 16.02 | 18.75 | 16.54 | 18.52 | 18.12 | 22.02 | 1.09 | .353 | .005 |
| Fellow workers not doing their jobs | 17.34 | 20.54 | 15.40 | 18.50 | 13.10 | 17.08 | 15.41 | 19.59 | 1.47 | .221 | .006 |
| Inadequate support by supervisor | 15.59 | 19.39 | 15.32 | 19.72 | 14.56 | 18.88 | 17.38 | 22.14 | .701 | .552 | .003 |
| Dealing with crisis situations | 16.90 | 18.57 | 16.53 | 17.45 | 17.47 | 19.18 | 16.91 | 18.57 | .093 | .964 | .001 |
| Lack of recognition for good work | 16.53 | 19.53 | 14.84 | 18.19 | 16.62 | 20.68 | 18.73 | 22.80 | 1.05 | .371 | 0,004 |
| Performing tasks not in job description | 17.76 | 21.36 | 14.35 | 17.49 | 13.39 | 16.65 | 15.76 | 18.40 | 1.65 | .176 | .007 |
| Inadequate or poor quality equipment | 19.10 | 24.12 | 16.95 | 22.28 | 14.06 | 18.15 | 14.58 | 18.96 | 1.91 | .127 | .008 |
| Assignment of increased responsibility | 28.00 | 26.95 | 27.03 | 25.53 | 24.45 | 24.16 | 28.42 | 25.65 | 1.03 | .378 | .004 |
| Periods of inactivity | 12.55 | 15.90 | 9.90 | 13.10 | 8.84 | 12.55 | 9.23 | 14.63 | 1.91 | .127 | .008 |
| Difficulty getting along with supervisor | 13.25 | 17.97 | 11.22 | 17.34 | 10.87 | 16.64 | 12.40 | 18.74 | .593 | .620 | .003 |
| Experiencing negative attitudes toward the organization | 10.13 | 16.70 | 11.04 | 17.44 | 8.74 | 15.93 | 9.82 | 16.82 | .666 | .573 | .003 |
| Insufficient personnel to adequately handle an assignment | 13.75 | 19.32 | 13.01 | 17.45 | 11.09 | 14.64 | 13.92 | 18.90 | 1.18 | .317 | .005 |
| Making critical on-the-spot decisions | 19.45 | 21.93 | 19.56 | 21.37 | 16.78 | 18.60 | 18.61 | 21.41 | .796 | .496 | .003 |
| Personal insult from customer / consumer / colleague | 14.59 | 21.24 | 12.04 | 17.62 | 10.34 | 16.85 | 10.06 | 17.97 | 1.78 | .151 | .008 |
| Lack of participation in policy-making decisions | 13.59 | 17.724 | 11.85 | 17.00 | 11.16 | 14.65 | 12.52 | 17.62 | .608 | .610 | .003 |
| Inadequate salary | 18.68 | 24.76 | 18.11 | 22.66 | 19.69 | 25.19 | 19.53 | 25.23 | .168 | .918 | .001 |
| Competition for advancement | 16.83 | 23.93 | 13.35 | 17.30 | 13.14 | 19.41 | 14.10 | 21.58 | .894 | .444 | .004 |
| Poor or inadequate supervision | 9.41 | 14.71 | 10.61 | 16.91 | 10.63 | 17.37 | 13.96 | 21.32 | 1.86 | .135 | .008 |
| Noisy work area | 12.55 | 18.18 | 13.93 | 20.41 | 13.16 | 20.86 | 12.41 | 20.22 | .191 | .903 | .001 |
| Frequent interruptions | 14.26 | 20.07 | 15.94 | 21.91 | 13.19 | 17.98 | 14.11 | 18.87 | .676 | .567 | .003 |
| Frequent changes from boring to demanding duties | 14.42 | 17.14 | 14.04 | 19.26 | 11.98 | 16.98 | 12.20 | 17.33 | .817 | .485 | .003 |
| Excessive paperwork | 20.65 | 23.17 | 20.18 | 22.55 | 15.79 | 19.26 | 20.43 | 23.87 | 2.38 | .068 | .010 |
| **Meeting deadlines** | 31.06 | 28.06 | 26.93 | 25.74 | 22.38 | 23.09 | 24.45 | 23.87 | 3.05 | **.029** | .014 |
| Insufficient personal time | 19.38 | 24.29 | 17.83 | 22.66 | 15.40 | 20.60 | 17.64 | 23.55 | .934 | .424 | .004 |
| Covering work for another employee | 17.36 | 20.80 | 18.73 | 22.48 | 15.13 | 18.64 | 16.08 | 20.12 | 1.15 | .328 | .005 |
| Poorly motivated coworkers | 18.34 | 21.78 | 18.02 | 21.28 | 17.62 | 21.02 | 18.53 | 22.66 | .069 | .976 | .001 |
| **Conflicts with other department** | 9.74 | 14.07 | 10.93 | 15.89 | 7.07 | 11.18 | 10.27 | 17.64 | 3.54 | **.015** | .012 |

*Note.* Welch’s F test was performed to identify work experience differences. η^2^ = eta-squared effect size; small: η^2^≥0.01; medium: η^2^≥0.06; large: η^2^≥0.14 [1].

**Supplementary Table 3**

*Descriptive Statistics and Results of ANOVAs on Educational Level Differences for all JSS Items*

| **Educational**  **Level** | **Vocational degree** | | **Undergraduate degree** | | **Postgraduate degree** | |  |  |  |
| --- | --- | --- | --- | --- | --- | --- | --- | --- | --- |
|  | (n = 18) | | (n = 567) | | (n = 117) | |  |  |  |
|  | ***M*** | ***SD*** | ***M*** | ***SD*** | ***M*** | ***SD*** | ***F/*** ***Welch’s F*** | ***p*** | ***η^2^*** |
| **Assignment of disagreeable duties** | 18.00 | 18.10 | 15.06 | 17.57 | 20.23 | 18.47 | 3.93 | **.027** | .012 |
| **Working overtime** | 17.44 | 18.52 | 15.23 | 20.17 | 24.73 | 24.14 | 7.81 | **.001** | .028 |
| **Lack of opportunity for advancement** | 15.17 | 13.66 | 16.33 | 21.09 | 25.05 | 25.05 | 6.33 | **.004** | .022 |
| Assignment of new or unfamiliar duties | 22.56 | 25.57 | 16.56 | 19.40 | 20.19 | 20.75 | 2.27 | .104 | .006 |
| Fellow workers not doing their jobs | 21.44 | 25.14 | 14.05 | 18.36 | 17.81 | 18.49 | 3.14 | .044 | .009 |
| **Inadequate support by supervisor** | 13.06 | 19.12 | 14.17 | 18.86 | 22.95 | 23.73 | 7.09 | **.002** | .027 |
| **Dealing with crisis situations** | 19.11 | 12.27 | 16.13 | 18.30 | 21.00 | 19.73 | 3.26 | **.048** | .010 |
| **Lack of recognition for good work** | 27.28 | 28.89 | 15.42 | 19.90 | 21.25 | 20.93 | 5.01 | **.011** | .018 |
| Performing tasks not in job description | 18.11 | 24.89 | 14.24 | 17.79 | 17.41 | 18.20 | 1.79 | .167 | .005 |
| Inadequate or poor quality equipment | 18.33 | 20.46 | 14.97 | 19.87 | 18.48 | 22.78 | 1.59 | .204 | .005 |
| Assignment of increased responsibility | 37.67 | 30.18 | 25.57 | 25.27 | 29.85 | 24.16 | 2.69 | .079 | .009 |
| Periods of inactivity | 14.89 | 19.14 | 9.27 | 13.07 | 11.31 | 15.91 | 2.35 | .096 | .007 |
| **Difficulty getting along with supervisor** | 7.28 | 8.11 | 11.04 | 17.13 | 15.53 | 19.92 | 4.83 | **.012** | .011 |
| Experiencing negative attitudes toward the organization | 13.44 | 20.11 | 9.17 | 16.22 | 12.15 | 17.88 | 2.02 | .134 | .006 |
| **Insufficient personnel to adequately handle an assignment** | 12.00 | 18.38 | 11.79 | 16.70 | 16.98 | 18.84 | 3.77 | **.031** | .013 |
| Making critical on-the-spot decisions | 28.50 | 29.94 | 17.34 | 19.75 | 21.46 | 21.86 | 2.84 | .070 | .012 |
| Personal insult from customer / consumer / colleague | 15.39 | 25.53 | 10.78 | 17.80 | 13.32 | 17.89 | 1.44 | .239 | .004 |
| **Lack of participation in policy-making decisions** | 13.56 | 19.19 | 11.14 | 15.81 | 16.11 | 18.52 | 3.68 | **.034** | .013 |
| **Inadequate salary** | 17.56 | 24.81 | 17.95 | 24.06 | 24.97 | 25.87 | 3.62 | **.035** | .011 |
| Competition for advancement | 7.83 | 9.53 | 13.76 | 20.58 | 16.02 | 19.53 | 1.46 | .233 | .004 |
| Poor or inadequate supervision | 11.67 | 19.03 | 10.87 | 17.65 | 13.15 | 19.57 | .774 | .461 | .002 |
| Noisy work area | 14.89 | 20.21 | 12.84 | 20.64 | 13.88 | 17.88 | .204 | .816 | .001 |
| Frequent interruptions | 10.89 | 19.49 | 13.79 | 19.13 | 16.97 | 21.22 | 1.56 | .210 | .004 |
| Frequent changes from boring to demanding duties | 15.33 | 20.53 | 12.19 | 17.52 | 15.97 | 17.68 | 2.40 | .091 | .007 |
| **Excessive paperwork** | 18.11 | 29.62 | 17.37 | 20.88 | 25.53 | 24.48 | 5.57 | **.007** | .019 |
| Meeting deadlines | 20.06 | 21.53 | 24.39 | 24.50 | 30.52 | 26.49 | 3.38 | .034 | .010 |
| Insufficient personal time | 16.06 | 20.31 | 16.25 | 22.15 | 21.68 | 23.66 | 2.88 | .057 | .008 |
| **Covering work for another employee** | 14.67 | 20.48 | 15.48 | 19.87 | 22.19 | 21.71 | 4.76 | **.014** | .015 |
| Poorly motivated coworkers | 19.67 | 25.05 | 17.09 | 21.04 | 22.47 | 23.15 | 3.09 | .046 | .009 |
| Conflicts with other department | 11.22 | 20.30 | 9.03 | 14.75 | 9.77 | 13.27 | .298 | .742 | .001 |

*Note.* Welch’s F test was performed to identify educational level differences. η^2^ = eta-squared effect size; small: η^2^≥0.01; medium: η^2^≥0.06; large: η^2^≥0.14 [1].

**References**

1. Cohen J. Statistical power analysis for the behavioral sciences. 2nd ed. NJ: Lawrence Erlbaum Associates; 1988.
